# Effect of freshwater mussels on the vertical distribution of anaerobic ammonia oxidizers and other nitrogen-transforming microorganisms in upper Mississippi river sediment

**DOI:** 10.7717/peerj.3536

**Published:** 2017-07-12

**Authors:** Ellen M. Black, Michael S. Chimenti, Craig L. Just

**Affiliations:** 1 Department of Civil and Environmental Engineering, University of Iowa, Iowa City, IA, United States of America; 2 Iowa Institute of Human Genetics, University of Iowa, Iowa City, IA, United States of America

**Keywords:** Freshwater mussels, Anaerobic ammonia oxidizers, Anammox, Nitrogen cycle, Sediment microbiology

## Abstract

Targeted qPCR and non-targeted amplicon sequencing of 16S rRNA genes within sediment layers identified the anaerobic ammonium oxidation (anammox) niche and characterized microbial community changes attributable to freshwater mussels. Anammox bacteria were normally distributed (Shapiro-Wilk normality test, *W*-statistic =0.954, *p* = 0.773) between 1 and 15 cm depth and were increased by a factor of 2.2 (*p* < 0.001) at 3 cm below the water-sediment interface when mussels were present. Amplicon sequencing of sediment at depths relevant to mussel burrowing (3 and 5 cm) showed that mussel presence reduced observed species richness (*p* = 0.005), Chao1 diversity (*p* = 0.005), and Shannon diversity (*p* < 0.001), with more pronounced decreases at 5 cm depth. A non-metric, multidimensional scaling model showed that intersample microbial species diversity varied as a function of mussel presence, indicating that sediment below mussels harbored distinct microbial communities. Mussel presence corresponded with a 4-fold decrease in a majority of operational taxonomic units (OTUs) classified in the phyla Gemmatimonadetes, Actinobacteria, Acidobacteria, Plantomycetes, Chloroflexi, Firmicutes, Crenarcheota, and Verrucomicrobia. 38 OTUs in the phylum Nitrospirae were differentially abundant (*p* < 0.001) with mussels, resulting in an overall increase from 25% to 35%. Nitrogen (N)-cycle OTUs significantly impacted by mussels belonged to anammmox genus *Candidatus* Brocadia, ammonium oxidizing bacteria family Nitrosomonadaceae, ammonium oxidizing archaea genus *Candidatus* Nitrososphaera, nitrite oxidizing bacteria in genus *Nitrospira*, and nitrate- and nitrite-dependent anaerobic methane oxidizing organisms in the archaeal family “ANME-2d” and bacterial phylum “NC10”, respectively. Nitrosomonadaceae (0.9-fold (*p* < 0.001)) increased with mussels, while NC10 (2.1-fold (*p* < 0.001)), ANME-2d (1.8-fold (*p* < 0.001)), and *Candidatus* Nitrososphaera (1.5-fold (*p* < 0.001)) decreased with mussels. Co-occurrence of 2-fold increases in *Candidatus* Brocadia and *Nitrospira* in shallow sediments suggests that mussels may enhance microbial niches at the interface of oxic–anoxic conditions, presumably through biodeposition and burrowing. Furthermore, it is likely that the niches of *Candidatus* Nitrososphaera and nitrite- and nitrate-dependent anaerobic methane oxidizers were suppressed by mussel biodeposition and sediment aeration, as these phylotypes require low ammonium concentrations and anoxic conditions, respectively. As far as we know, this is the first study to characterize freshwater mussel impacts on microbial diversity and the vertical distribution of N-cycle microorganisms in upper Mississippi river sediment. These findings advance our understanding of ecosystem services provided by mussels and their impact on aquatic biogeochemical N-cycling.

## Introduction

Native freshwater mussels (Order Unionida) are ecosystem engineers that significantly alter benthic habitats through biodeposition of feces and pseudofeces, rich in ammonium (NH_4_^+^) and organic carbon (C), into sediment (Thorp et al., 1998; Vaughn & Hakenkamp, 2001; Bril et al., 2014). The estimated mussel filtration capacity in a 480 km, Upper Mississippi River (UMR) segment, as a percentage of river discharge, is up to 1.4% at high flows, up to 4.4% at moderate flows and up to 12.2% during low flows (Newton et al., 2011). The mussels in this river segment collectively filter over 14 billion gallons of water, remove tons of biomass from the overlying water, and deposit tons of reduced C and nitrogen (N) at the water-sediment interface each day (Newell, 2004). The pocketbook mussel (*Lampsilis cardium*) and threeridge mussel (*Amblema plicata*) comprise up to 38% and 56% of the mussel biomass in the UMR, respectively (Newton et al., 2011). A habitat near Buffalo, Iowa, in UMR Pool 16, had mean densities of 1.56 *L. cardium*-m^−2^ and 7.18 *A. plicata*-m^−2^ that correlated with fine sediment diameters (*d*_50_ = 0.300 ± 0.121 mm) which were presumably influenced by mussel burrowing (Young, 2006). Mussels live primarily buried in sediment, with their posterior end often flush with the sediment surface (Haag, 2012), or slightly below the surface in soft sediments (Allen & Vaughn, 2009; Allen, 1923; Matteson, 1955). This positions adult freshwater mussels 6–10 cm into the sediment with tendencies toward more shallow burrowing during the spring and summer (Schwalb & Pusch, 2007). Extensive observations in the UMR concluded that *A. plicata* were often found with portions of their shell above the water-sediment interface, while *L. cardium* burrow a few cm into the sediment during the summer (Newton, Zigler & Gray, 2015). Additionally, *A. plicata* often burrowed up to 2.5 cm vertically (Allen & Vaughn, 2009) in response to stressors while *L. cardium* moved more horizontally when stressed (Newton, Zigler & Gray, 2015). Two common stressors, that happen to be created by the mussels themselves, are low dissolved oxygen (DO) and elevated ammonia (NH_3_) and NH_4_^+^ (Bril et al., 2017; Haag, 2012). We hypothesize that this frequent vertical and horizontal movement by mussels, many times as an indirect and/or direct response to their own waste production, has a significant impact on porewater chemistry and microbiology in UMR sediments.

The evidence for freshwater mussel impacts on aquatic chemistry is compelling, especially for nutrients. A dense mussel population can sequester 2 g C day^−1^m^−2^, 200 mg N day ^−1^m^−2^, and 50 mg phosphorus day^−1^m^−2^ from river water into sediment (Strayer, 2014). During the summer months, biodeposition-derived N from mussels was roughly 67% NH_4_^+^, 28% amino acids, and 5% urea (Bayne, 1973). Mussel biodeposition accounted for up to 40% of total N demand in freshwaters and up to 74% of N in the food web, but was sometimes dampened (Atkinson, Kelly & Vaughn, 2014) in high nutrient environments (Atkinson, Kelly & Vaughn, 2014). Our previous work showed mussel burrowing and biodeposition, just below the water-sediment interface, increased porewater NH_4_^+^, nitrate (NO_3_^−^), nitrite (NO_2_^−^), and total organic C concentrations by 160%, 38%, 40%, and 26%, respectively (Bril et al., 2017; Bril et al., 2014). However, the experimental design of our previous work limited our ability to assess the effects of mussels on the broad microbial community that was transforming N simultaneously and, quite likely, synergistically.

**Figure 1:**
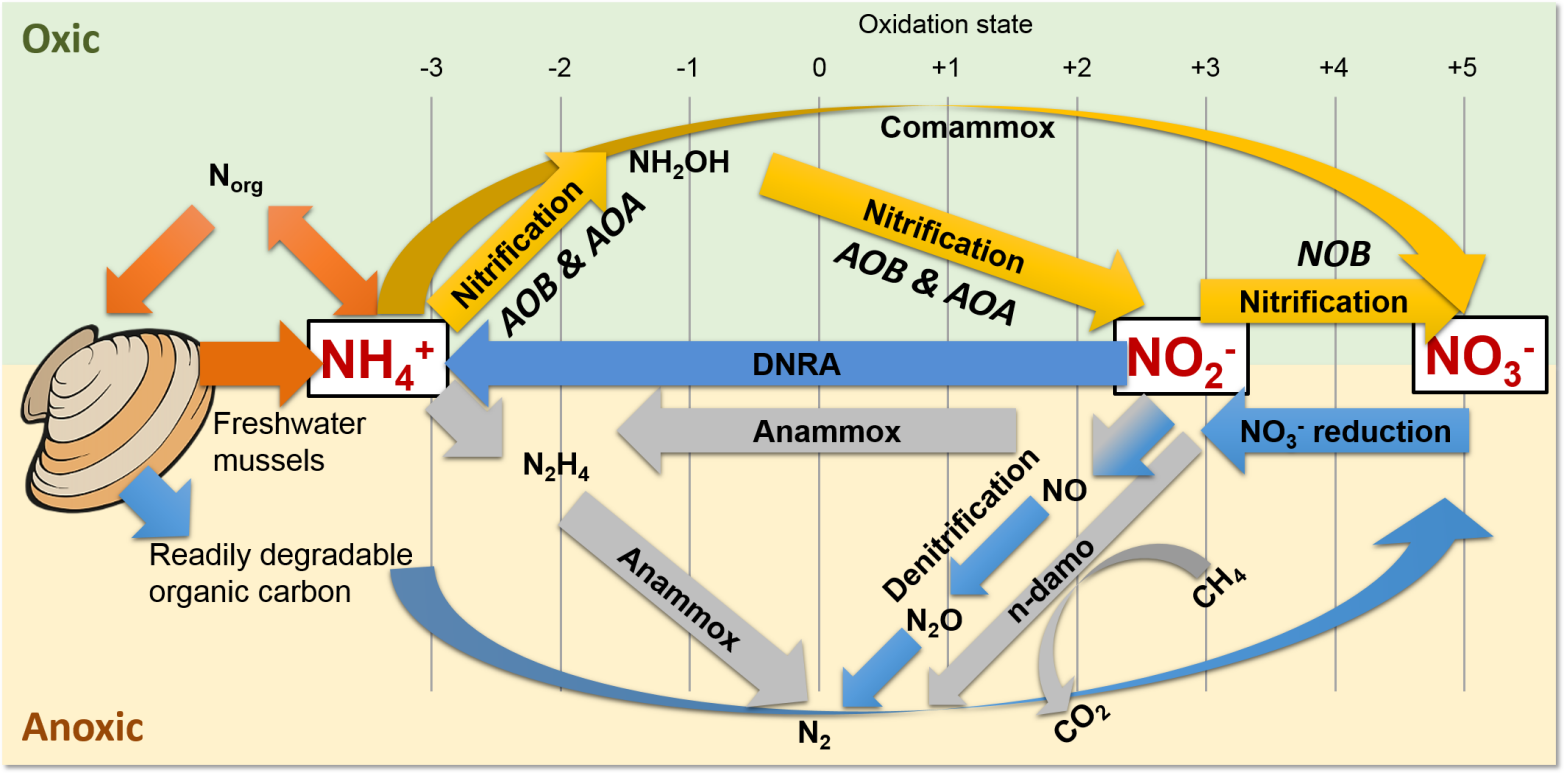
Freshwater mussels deposit feces and pseudofeces containing nitrogen and carbon at the water-sediment interface (i.e., oxic-anoxic transition). NH_4_^+^ resulting from mussel biodeposits may be oxidized via nitrification and comammox in oxic conditions (yellow arrows), and/or by anammox and n-damo near the oxic-anoxic interface (gray arrows). Oxidized nitrogen species (NO_2_^−^ and NO_3_^−^) may be reduced by dissimilatory nitrate reduction to ammonium (DNRA) and denitrification pathways (blue).

The UMR is an N-rich agro-ecosystem (Hill et al., 2011; Hill et al., 2008; Houser & Richardson, 2010; Ikenberry et al., 2014; Schilling, Wolter & McLellan, 2015) shown to foster high microbial N transformations (Mason et al., 2016; Millar et al., 2015) potentially making the effects of mussels on a variety of N-transforming bacteria and archaea more pronounced than in any other freshwater environment. The first step in transforming biologically active N is nitrification by aerobic ammonium oxidizing bacteria (AOB) (Fig. 1, yellow arrows), such as the genera *Nitrosomonas* and *Nitrosospira* in the Nitrosomonadaceae family (Aakra et al., 2001; Burrell, Phalen & Hovanec, 2001; Hayatsu, Tago & Saito, 2008; Prosser, Head & Stein, 2014), and aerobic ammonium oxidizing archaea (AOA) in multiple candidate genera. AOB and AOA are metabolically diverse (Leininger et al., 2006) and serve a functionally important role of catalyzing the rate limiting step of nitrification (Martens-Habbena et al., 2009) in various freshwater niches. For example, *Candidatus* Nitrososphaera (phylum Thaumarchaetoa), a group of thermophilic AOA (Hatzenpichler et al., 2008), and AOB species in genera *Nitrosospira* and *Nitrosococcus* (Koper et al., 2004) can use urea as an alternative source of NH_4_^+^ (Spang et al., 2012). AOA often outnumber AOB (Prosser & Nicol, 2008) due to their ability to grow at NH_4_^+^ concentrations below 10 nM (Martens-Habbena et al., 2009), compared to 10 µM for some AOB species (Bollmann, Bar-Gilissen & Laanbroek, 2002). In the second step of nitrification, nitrite oxidizing bacteria (NOB), such as *Nitrospira* (phylum Nitrospirae), and *Nitrobacter*, *Nitrococcus*, and *Nitrospina* (phylum Proteobacteria) (Prosser, Head & Stein, 2014), aerobically oxidize NO_2_^−^ to NO_3_^−^ (Fig. 1, yellow arrows).

*Nitrospira* are the most abundant and diverse group of NOB and dominate numerous habitats, ranging from freshwater sediment to engineered wastewater treatment plants (Daims et al., 2015; Koch et al., 2015; Lucker et al., 2010). Furthermore, NOB species *Nitrospira moscoviensis* and *Nitrospira lenta* can derive NH_4_^+^ from urea hydrolysis, provide NH_4_^+^ to AOB, and subsequently may oxidize NO_2_^−^ from AOB in a process deemed “reciprocal feeding of nitrifiers” (Daims et al., 2015; Koch et al., 2015). *Candidatus* Nitrospira inopinata, can also use urea as an alternative NH_4_^+^ source (Daims et al., 2015) and has all the genes necessary for complete ammonia oxidation (comammox) to NO_3_^−^ (Van Kessel et al., 2015) (Fig. 1, yellow curved arrow). Denitrifiers complete the conventional N-cycle by sequentially reducing NO_3_^−^ to nitric oxide (NO), nitrous oxide (N_2_O), and nitrogen gas in anoxic and high C environments (Fig. 1, blue arrows) (Hayatsu, Tago & Saito, 2008).

A specialized group of bacteria in the phylum Planctomycetes, anaerobic ammonium oxidizing (anammox) bacteria, oxidize NH_4_^+^, utilize NO_2_^−^ as a terminal electron acceptor, and produce N_2_ gas (Kartal et al., 2011; Kuenen, 2008) (Fig. 1, gray arrows). Anammox bacteria thrive at the interface of oxic–anoxic conditions due to dependence on NO_2_^−^ production by AOB or AOA (Thamdrup, 2012). Anammox and NOB compete for NO_2_^−^ in low substrate environments, and this is especially true for *Nitrospira* NOB, which share a homologous form of the key enzyme catalyzing NO_2_^−^ oxidation with anammox (Lucker et al., 2010). In another example, *N. moscoviensis* can adapt to a range of oxygen concentrations by coupling formate oxidation and NO_3_^−^ reduction (Koch et al., 2015). Recently, an N-cycling enrichment culture revealed comammox bacteria co-occurring with anammox bacteria in the genus *Candidatus* Brocadia, presumably enhanced by the ability of commamox organisms to oxidize NH_4_^+^ in low oxygen conditions (<3.1 µM) (Van Kessel et al., 2015). *Nitrospira* species, *N. moscoviensis* and “*Ca.* Nitrospira inopinata” in particular, are examples of NOB which harbor a unique ability to assist or compete with anammox for N-substrate in a variety of niches (Lucker et al., 2010).

Shallow sediments also pose a competitive niche for anammox bacteria because of high NH_4_^+^ fluxes into oxic sediment and NO_2_^−^ limitations from denitrification (Thamdrup, 2012; Trimmer & Engstrom, 2011). Another addition to the suite of known N-transformations includes prokaryotic coupling of anaerobic oxidation of methane with denitrification (Raghoebarsing et al., 2006). In nitrite- and nitrate-dependent anaerobic methane (CH_4_) oxidation (n-damo) (Raghoebarsing et al., 2006; Thamdrup, 2012; Welte et al., 2016), NO_3_^−^ reduction to NO_2_^−^ and NO_2_^−^ reduction to N_2_ are coupled with CH_4_ oxidation to CO_2_ (Fig. 1, gray line and curved arrows). Nitrate-damo biochemical processes have been linked to family “ANME-2d” (Ding et al., 2016; Haroon et al., 2013), while nitrite-damo was discovered for “*Candidatus* Methylomirabilis oxyfera”(Ettwig et al., 2010) in phylum “NC10” (Ettwig et al., 2009; Luesken et al., 2011; Padilla et al., 2016), and both are widespread in anoxic freshwater sediments (Deutzmann & Schink, 2011; Ding et al., 2016; Ettwig et al., 2009; Hu et al., 2009).

Mollusks have been shown to influence the diversity of microbial communities and abundance of N-transforming microorganisms. For example, metagenomic profiling revealed a marine California mussel (*Mytilus californianus*) shell provided a niche for N- and C-transforming microorganism populations (Pfister, Meyer & Antonopoulos, 2010), and a restored oyster reef enhanced nitrification and denitrification rates greater than 10-fold (Kellogg et al., 2013). Furthermore, an experimental microcosm study reported enhanced prokaryotic metabolic activity and diversity following a biodeposition rate of 10 g m^−2^ d^−1^ of mussel feces and pseudofeces (Pollet et al., 2015). Additionally, clusters of the zebra mussel (*Dreissena polymorpha*) in a lake increased heterotrophic bacteria density, activity, and diversity (Lohner et al., 2007). Since the impact of native freshwater mussels on prokaryotic diversity and abundance in the UMR is largely unknown, this study utilized targeted and non-targeted sequencing of the 16S rRNA gene to determine how N-transforming microorganisms and microbial community structure differs in sediments with mussels compared to sediments without mussels.

## Materials and Methods

UMR sediments were collected within a dense, well-characterized mussel assemblage in the Buffalo Habitat of UMR Pool 16 (Young, 2006) (41.452804, −90.763299), and from a slightly up-river location with no mussels (41.451540, −90.753275) using a 3-inch diameter, hammer-driven, acrylic tube (Batch 1 samples) or a 2-inch diameter, post-driver sediment sampler with a polypropylene liner (Multi-Stage Sediment Sampler, Batch 2 samples; Art’s Manufacturing and Supply, Inc., American Falls, ID, USA). Batch 1 sediment was used to identify the vertical distribution of anammox bacteria below freshwater mussels. For Batch 1, the acrylic tube for each core (*n* = 3 with-mussels) was penetrated at 1, 3, 5, 7, 11, and 15 cm sediment depths with a 3/8th-inch diameter, ethanol flame-sterilized drill bit to enable sediment collection. In comparison, Batch 2 sediment was used to characterize anammox abundance, microbial diversity, and community structure in shallow sediments below mussels. For Batch 2, the polypropylene liner for each sediment core (*n* = 5 with-mussels, *n* = 5 no-mussels) was penetrated at depths of 3 cm and 5 cm. Sediment was sampled for DNA isolation (in quadruplicate) for a combined sample size of *n* = 20 for 3 cm depth with-mussels, *n* = 20 for 5 cm depth with-mussels, *n* = 20 for 3 cm depth without mussels, and *n* = 20 for 5 cm depth without mussels. Genomic DNA was isolated from 0.25 g of each sediment sample (PowerSoil® DNA Isolation Kit; MoBio Laboratories, Inc., Carlsbad, CA, USA) and stored at −20°C. Batch 2 Genomic DNA was used for anammox-targeted qPCR (*n* = 20 for each treatment) and 16S rRNA gene amplicon sequencing (*n* = 10 for each treatment).

### Anammox 16S rRNA gene quantification

Microbial culture from a sidestream deammonification process (Hampton Roads Sanitation District, Virginia Beach, VA, USA) served as a source of anammox genetic material for qPCR standard curve construction. PCR products (primers A483f (5′-GTCRGGAGTTADGAAATG-3′) and A684r (5′-ACCAGAAGTTCCACTCTC-3′) (Sonthiphand & Neufeld, 2013)) of the anammox 16S rRNA gene was purified with Qiaquick PCR purification Kit (Qiagen Inc.; Valencia, CA, USA), and cloned into the pCR 2.1-TOPO® vector using the TOPO® TA cloning Kit (Invitrogen Corp.; Carlsbad, CA, USA). Clones were Sanger sequenced at the University of Iowa Institute of Human Genetics with M13F (5′-TGTAAAACGACGGCCAGT-3′) and M13R (5′-CAGGAAACAGCTATGAC-3′) primers to ensure anammox 16S rRNA PCR products were inserted into the vector. Nucleotide sequences were aligned using the Standard Nucleotide Basic Local Alignment Search Tool (Altschul et al., 1997) (GenBank Accession: KU047953) and classified as *Candidatus* Brocadiales (of the Planctomycetes phylum) with a 95% confidence threshold using RDP Naïve Bayesian rRNA Classifier Version 2.10 (Wang et al., 2007). Plasmid DNA concentration was quantified with Qubit® Fluorometer 1.0 (Thermo Fisher Scientific, Inc.; Waltham, MA, USA), serially diluted, and used to construct qPCR calibration curves.

The anammox 16S rRNA gene from batches 1 and 2 was quantified (Wang et al., 2015) with qPCR using QuantStudio™ 7 Flex Real-Time PCR System (Thermo Fisher Scientific, Inc.; Waltham, MA, USA) with primers A483f and A684r (Sonthiphand & Neufeld, 2013) and analyzed with QuantStudio™ Real-Time PCR Software (Thermo Fisher Scientific, Inc.; Waltham, MA, USA). The threshold cycle (C_*t*_) curves were satisfactory (slope = − 3.374, *Y*-int = 36.702, *R*^2^ = 0.998, and amplification efficiency = 97.99%), and PCR product dissociation curves revealed singe peaks centered at a melting temperature of 83°C. The statistical significance of 16S rRNA gene copies was determined via a one-way, repeated measures analysis of variance (ANOVA) (SigmaPlot 13.0, Systat Software, Inc., Chicago, IL, USA) between the 4 treatment groups (*n* = 20) following a passed normality test (*p* = 0.826, Shapiro–Wilk) and an equal variance test (*p* = 0.073, Brown-Forsythe). Pairwise multiple comparison procedures were completed via the Holm-Sidak method with a significance level of 0.050 and a power of 0.990.

### Non-targeted amplicon sequencing of the 16S rRNA gene

Batch 2 genomic DNA (20 µL, 1–50 ng/µL) was analyzed by the Argonne National Laboratory, Environmental Sample Preparation and Sequencing Facility (ESPSF) utilizing the Earth Microbiome Project protocol (http://www.earthmicrobiome.org/emp-standard-protocols/16s/). All samples were analyzed together in one batch. The v4 region of prokaryotic 16S rRNA gene (515F-806R) was amplified using the following conditions: 3 min at 94°C, 35 cycles of 94°C for 45 s, 50°C for 60 s, and 72°C for 90 s, followed by 10 min at 72°C (Caporaso et al., 2012). The PCR mixture consisted of 13.0 µL PCR grade water, 10.0 µL 5 PRIME HotMasterMix (Quanta Biosciences, Beverly, MA, USA), 1.0 µL genomic DNA, and 0.5 µL forward and reverse primers (10 µM). 16S rRNA gene amplicon libraries were sequenced by ESPSF using Illumina MiSeq paired end reads (2 × 151 bp) (Caporaso et al., 2012) and uploaded to MG-RAST (ID’s: 4705672.3–4705709.3) and NCBI (BioProject ID PRJNA374585).

Determining the operational taxonomic units (“OTUs”) in each sample from the raw 16S rRNA gene amplicon reads was accomplished using the default Quantitative Insights into Microbial Ecology (QIIME) open-reference pipeline (Navas-Molina et al., 2013). Briefly, the QIIME open-reference pipeline takes paired-end reads as input, which are then joined, demultiplexed, filtered, and clustered into OTUs with uclust (Edgar, 2010). Representative sequences from each cluster were aligned (Caporaso et al., 2010) to GreenGenes 13.5 reference database (DeSantis et al., 2006) with a 97% similarity threshold. RDP classifier (Wang et al., 2007) was used for taxonomy assignment, PyNAST (Caporaso et al., 2010) was used for multiple sequence alignment. Phylogenetic trees were constructed using FastTree2.1.3 with default settings (Price, Dehal & Arkin, 2010). The OTU table from QIIME open reference picking (‘otu_table_mc2_w_tax_no_pynast_failures_json.biom’ in the standard QIIME workflow) was imported into R using the phyloseq package (McMurdie & Holmes, 2013) for downstream analysis, along with the corresponding phylogenetic tree (‘rep_set.tre’) and a metadata mapping file. These datasets were merged to create a single ‘physeq’ object representing the experiment. Alpha-diversity was calculated on the unfiltered OTU abundance data using the Observed species, Chao1 (Chao & Chiu, 2001), and Shannon (Li et al., 2011) metrics. Beta-diversity was calculated using a matrix of Bray-Curtis (Bray & Curtis, 1957) intersample distances and ordination plots calculated with non-metric multidimensional scaling (NMDS). Differential abundance analysis was carried out using the DESeq2 (Love, Huber & Anders, 2014) R package with default settings (test type was “Wald,” fit type was “parametric”). Translating physeq objects into a compatible DESeq2 object was performed with the “phyloseq_to_deseq2” function. The complete data analysis R script can be downloaded from the public GitHub repository: https://github.com/mchimenti/black_chimenti_just_phyloseq/blob/master/phyloseq.r.

Analysis at the OTU level provided a fine scale resolution for significant differences in microbial ecology between mussel and no mussel treatments. To put these results into a biological context, the genus-level OTU file was used to compare relative abundances for N-cycle phylotypes. These groups include AOA genus *Candidatus* Nitrososphaera, nitrate-damo family “ANME-2d”, NOB genus *Nitrospira*, anammox genus *Candidatus* Brocadia, AOB family Nitrosomonadaceae, and nitrite-damo phylum “NC10”. Relative abundance counts for each N-cycle group was tested for statistical significance between treatments, using metadata groups “3 cm with-mussels” (*n* = 10), “5 cm with-mussels” (*n* = 10), “3 cm no-mussels” (*n* = 10), and “5 cm no-mussels” (*n* = 10). 1-way ANOVA’s of each N-cycle group was performed using the Kruskal–Wallis test (*p* < 0.05) with Dunn’s multiple correction test (Padj < 0.05) (GraphPad Prism 7.0; La Jolla, CA, USA). Similarly, multiple comparisons were made between all N-cycle phylotype groups and their respective treatments (*n* = 10); significant differences between relative abundances were tested using the Kruskal–Wallis test (*P* < 0.0001) and Dunn’s multiple comparison test (Padj < 0.05).

## Results

### Anammox-targeted 16S rRNA gene quantification

**Figure 2:**
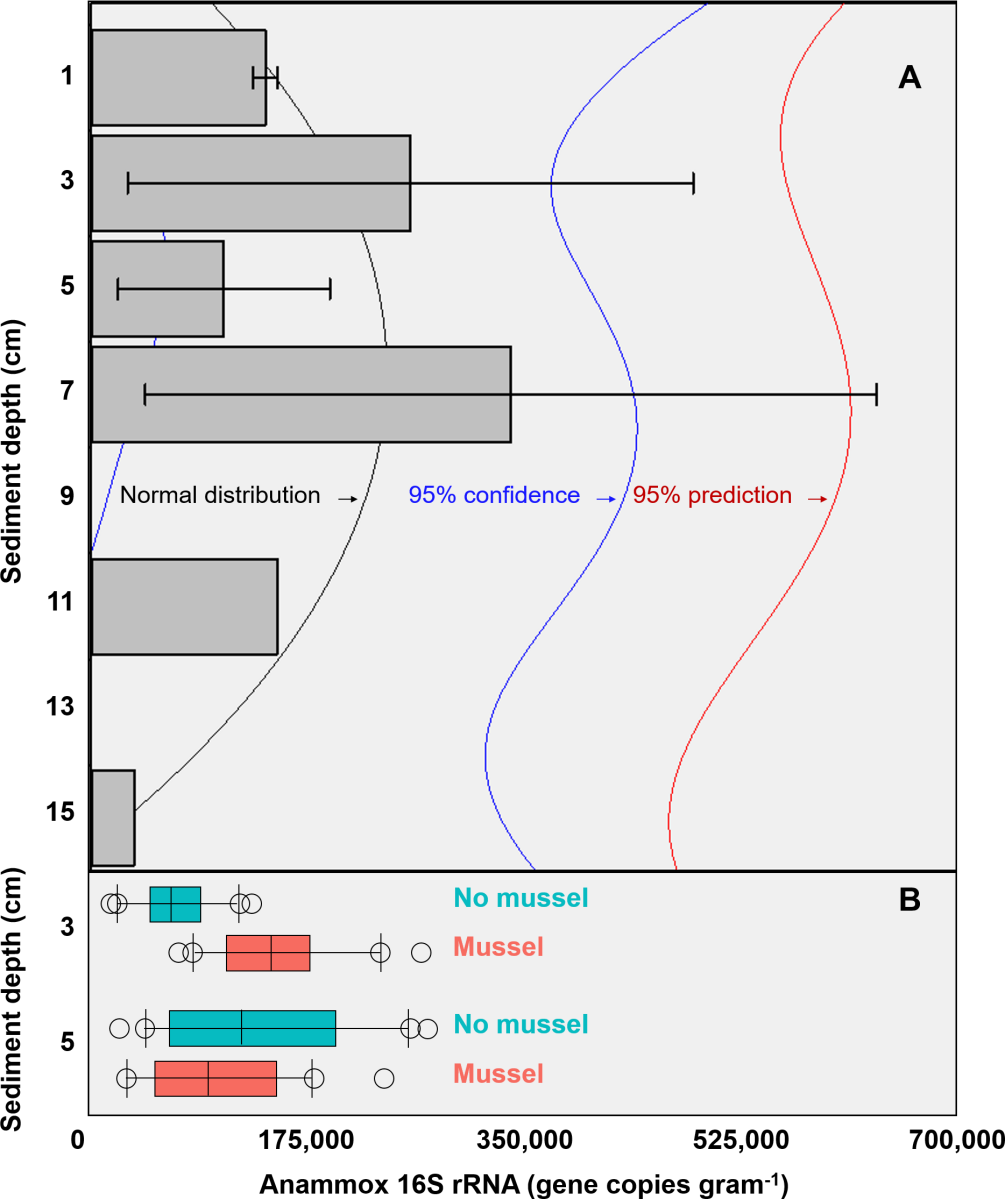
(A) The mean anammox 16S rRNA gene copies (per gram of sediment) in the presence of mussels were normally distributed (Shapiro–Wilk normality test, *W*-statistic =0.954, *p* = 0.773) with depth (Batch 1 data). Error bars represent 1 standard deviation from the mean. (B) Mussels (salmon-colored data) significantly increased the anammox 16S rRNA gene copies at 3 cm depth (*p* < 0.001; Batch 2 data). The anammox gene copies were statistically indistinguishable with mussels as compared to the no mussels (turquoise-colored data) sediments at 5 cm (Batch 2 data). The outer most open circles in Fig. 2B represent data outliers, box boundaries represent the 25th and 75th percentile, the line within the box is the median, and error bars indicate 10th and 90th percentiles.

The targeted 16S rRNA gene data from Batch 1 (*n* = 3, with-mussels) indicated an anammox bacterial gene copy maximum (∼3 ×10^5^ copies g^−1^ sediment) between 3 cm and 7 cm sediment depth in the presence of mussels (Fig. 2A). The Batch 1 data was normally distributed between 1 cm and 15 cm (Shapiro–Wilk normality test, *W*-statistic = 0.954, *p* = 0.773). Only one sediment core went beyond 7 cm leaving anammox bacterial gene copy data at 11 cm and 15 cm without replicates. The Batch 2 data (*n* = 20 for 3 cm with-mussels, *n* = 20 for 5 cm with-mussels, *n* = 20 for 3 cm no-mussels, *n* = 20 for 5 cm no-mussels) showed that anammox bacteria experienced a 2.2-fold increase (*p* < 0.001) at 3 cm with-mussels compared to the no-mussels control (Fig. 2B). The anammox gene copies measured at 5 cm were statistically indistinguishable between the with-mussels and no-mussels treatments.

### Non-targeted sequencing of the 16S rRNA gene

Summing across all samples, a total of 2,103,661 amplicon sequences were analyzed and about 76,000 unique OTUs were reported by QIIME. Of the unique OTUs, 18,777 had 10 or more reads and 3,916 OTUs had counts exceeding 100 reads. Mussel bed samples had read counts of 45,290 (±15,271) at 3 cm sediment depth and 52,451 (±7,044) at 5 cm sediment depth, while no-mussel samples had 48,920 (±7,517) read counts at 3 cm depth and 63,706 (±25,379) at 5 cm sediment depth (read depths depicted in Fig. S1). The top phyla in mussel bed sediments were Proteobacteria (40.7%), Nitrospirae (35.2%), Chloroflexi (5.9 %), Euryarchaeota (5.0%), Chlorobi (4.2%), and Bacteroidetes (2.3%). Proteobacteria decreased by about 6% with mussels while Nitrospirae increased by 10% with mussels. The most abundant taxonomic families in the Nitrospirae phylum were *Thermodesulfovibrionaceae* (55%), “FW” (33%), and Nitrospiraceae (13%), and were 5% less, 3% and 2% greater than in no-mussel samples, respectively. With mussels, Proteobacteria taxonomic classes consisted of the following proportions: 68% Deltaproteobacteria (8% less than without-mussels), 16% Gammaproteobacteria, and 15% Betaproteobacteria. A majority of these Deltaproteobacteria OTUs were from “BPC076”, Desulfarculales, and Syntrophobacterales taxanomic orders, while orders Burkholderiales and Xanthomonodales made up a majority of Betaproteobacteria and Gammaproteobacteria taxons.

**Figure 3:**
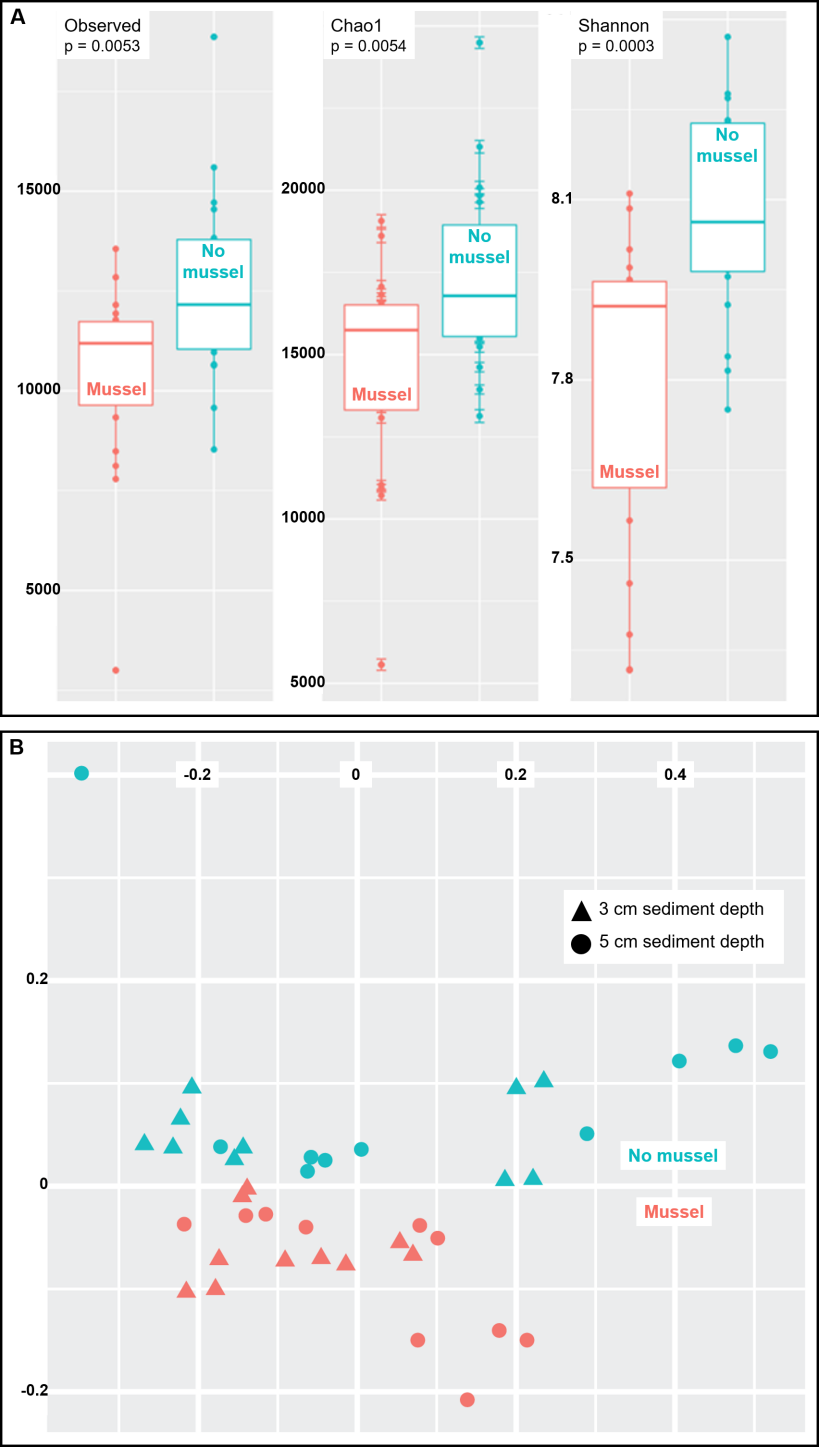
(A) Sediments with mussels have lower observed species richness (*p* = 0.005), Chao1 diversity (*p* = 0.005), and Shannon (*p* = 0.0003) diversity than no-mussel sediments. (B) NMDS analysis using Bray-Curtis distances revealed sample clustering as a function of mussel presence, but not sediment depth.

Species richness was analyzed using three common measures: Observed species, Chao1 and Shannon indices (*n* = 20 with-mussels and *n* = 20 without mussels). Together, the three measures indicated a decrease in microbial community richness and evenness in the presence of mussels as compared to sediments without mussels (Fig. 3A). The observed decrease in alpha-diversity reached significance for each of the three measures tested (*p* = 0.0054 or lower). A similar result was obtained when calculating alpha-diversity measures in samples exclusively from 3 cm (*n* = 10) or exclusively from 5 cm (*n* = 10) depths in the presence and absence of mussels. However, the decrease in richness was more pronounced at 5 cm than at 3 cm depth (Figs. S2 and S3).

To compare intersample diversity in species abundances and community composition (“beta diversity”), we employed NMDS scaling to accurately visualize, in 2D space, the higher-order community structure between with-mussels and no-mussels samples (Fig. 3B). The NMDS model produced an excellent representation of the bray–curtis distances for all samples (convergence in 20 iterations, stress ∼ 0.06; shepard plot shown in Fig. S4). The beta diversity clearly differentiated as a function of mussel presence, but not sediment depth (Fig. 3B). Taken together, these data show that mussel presence had a pronounced influence on the microbial community evenness, richness, and composition within the sediment.

Differential abundances in OTUs did not reach significance for metadata values of sediment depth or comparisons between sediment cores. On the other hand, there were numerous differences in OTU abundances when comparing sediment with mussels and without mussels. We performed a differential abundance estimation with the DESeq2 R package using mussel presence status (*n* = 20 with-mussels, *n* = 20 no-mussels) as our covariate. 734 OTUs (or 0.94% of the 77,288 OTUs tested) reached significance with a false discovery rate of 0.01. The vast majority of OTUs belonging to the phyla Gemmatimonadetes, Actinobacteria, Acidobacteria, Plantomycetes, Chloroflexi, Firmicutes, Crenarcheota, and Verrucomicrobia decreased by at least 4-fold in the presence of mussels. In contrast, Proteobacteria showed a marked decrease in order Alphaproteobacteria, while showing mixed increasing and decreasing OTUs among Beta-, Delta-, and Gammaproteobacteria. Phylum Nitrospirae also had 38 OTUs which were differentially abundant with *p*-adj < 0.001. OTUs assigned to the GreenGenes taxonomic family of “0319-6A21” were the most abundant among those OTUs increasing without mussels, while families *Thermodesulfovibrionaceae* and “FW” were most abundant among those OTUs increasing with mussels.

**Figure 4:**
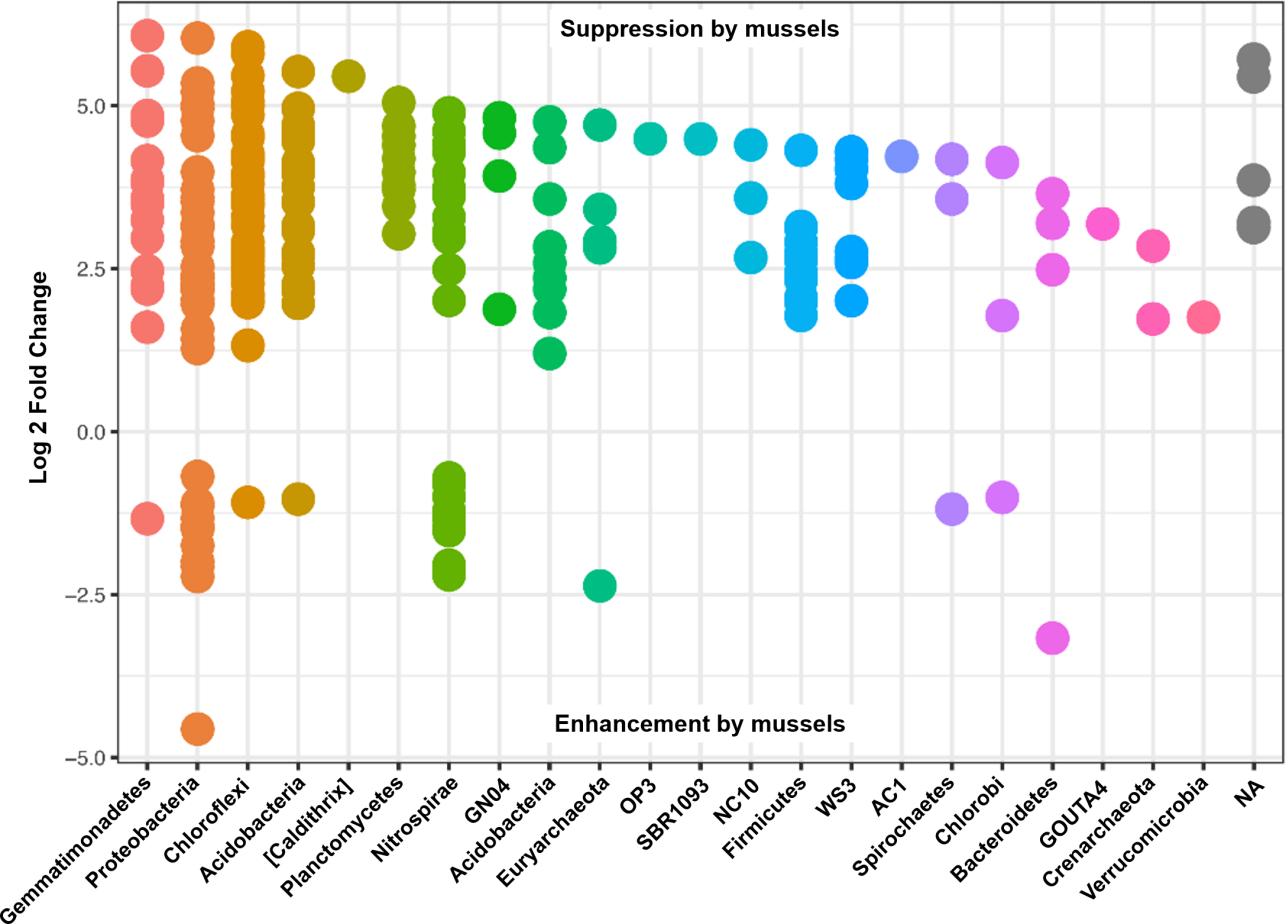
Results from a DESeq2 differential abundance analysis expressed as Log2FC comparison of with-mussels and no-mussels samples. Negative Log2FC represent phyla enhanced in the mussel bed and each point represents an individual OTU. To enhance clarity, only those OTUs with *p*-adj <0.0001 are shown.

Many of the Nitrospirae taxons that increased without mussels did so from a smaller average abundance (17 average counts for Nitrospira and up to 126 average counts for *Thermodesulfovibrionaceae*) relative to those that were increased with mussels (209 average counts for *Nitrospira* and up to 581 average counts for *Thermodesulfovibrionaceae*). This explains the 10% increase in Nitrospirae abundance when summing across all samples with mussels. Figure 4 shows the Log2FC categorized by phyla for OTUs with *p*-adj < 0.0001 (to enhance visual clarity). Significant differences within the Nitrospirae phylum were represented by increases of genus “HB118” in family *Thermodesulfovibrionaceae* (2.0Log2FC from a mean count of 52, *p* < 0.001) and unclassified *Nitrospira* species (0.8Log2FC from an average count of 209, *p* < 0.001) with mussels. No-mussel treatments showed increases in genus “LCP-6” from family *Thermodesulfovibrionaceae* (3.6Log2FC from an average count of 126, *p* < 0.001) and unclassified *Nitrospira* species (2.1Log2FC from an average count of 17, *p* < 0.001).

Despite seemingly even representation of phylum Thaumarchaeota between treatments, unclassified species from *Candidatus* Nitrososphaera were enhanced from an average abundance of 126 (1.73Log2FC, *p* < 0.001) without mussels, and AOA species, *Candidatus* Nitrososphaera gargensis increased from an average count of 16 (2.85Log2FC, *p* < 0.001) without mussels. One OTU classified in the anammox genus, *Candidatus* Brocadia, increased from an average count of 17 (3.72Log2FC, *p* < 0.001) without mussels, while another OTU classified as an unknown *Candidatus* Brocadia species increased from a mean count of 16 (1.2Log2FC, *p* = 0.001) with-mussels. Furthermore, OTUs belonging to the AOB family Nitrosomonadaceae increased from an average abundance of 6 (1.9Log2FC, *p* < 0.001) with mussels. Without mussels, taxonomic groups capable of nitrite-damo, phylum “NC10”, increased from average abundances up to 130 (4.4 Log2FC, *p* < 0.001), and nitrate-damo family “ANME-2d” increased from average abundances up to 59 (3.4 Log2FC, *p* < 0.001). A summary of Log2FC values for OTUs relevant to N-transformations are listed in Table S1.

N-cycle phylotypes were examined for statistically significant relative abundances between treatments of mussel presence and sediment depth (Table 1). *Candidatus* Nitrososphaera experienced a 2.6-fold decrease (*p* = 0.047) with mussels at 5 cm sediment depth. ANME-2d was three times greater (*p* = 0.049) at 5 cm sediment depth without mussels, compared to 3 cm sediment depth without mussels. Within the mussel bed, *Nitrospira* were 1.7 times greater (*p* = 0.0497) at 3 cm depth, and experienced a 1.9-fold increase (*p* = 0.025) with mussels at 3 cm sediment depth versus control. *Candidatus* Brocadia was three times greater (*p* = 0.013) at 5 cm depth without mussels versus 3 cm without mussels, and the 3 cm sediment showed a 2-fold increase (*p* = 0.002) with mussels versus control. Nitrosomonadaceae was 2.7 times greater (*p* = 0.015) at 3 cm with mussels versus 5 cm depth with mussels.

**Table 1:** The percent relative abundance of N-cycle organisms for mussel and depth treatments.

Taxonomic classification	N-cycle classification	Mean percent relative abundance			
		3 cm with-mussels	3 cm no-mussels	5 cm with-mussels	5 cm no-mussels
*Candidatus* Nitrososphaera	AOA	0.26	0.44	0.22	0.58
ANME-2D	Nitrate-damo	0.12	0.21	0.11	0.63
NC10	Nitrite-damo	0.0039	0.02	0.0035	0.08
*Nitrospira*	NOB/comammox	1.92	1.00	1.11	0.85
*Candidatus* Brocadia	Anammox	0.10	0.05	0.07	0.15
Nitrosomonadaceae	AOB	0.27	0.13	0.10	0.08

**Figure 5:**
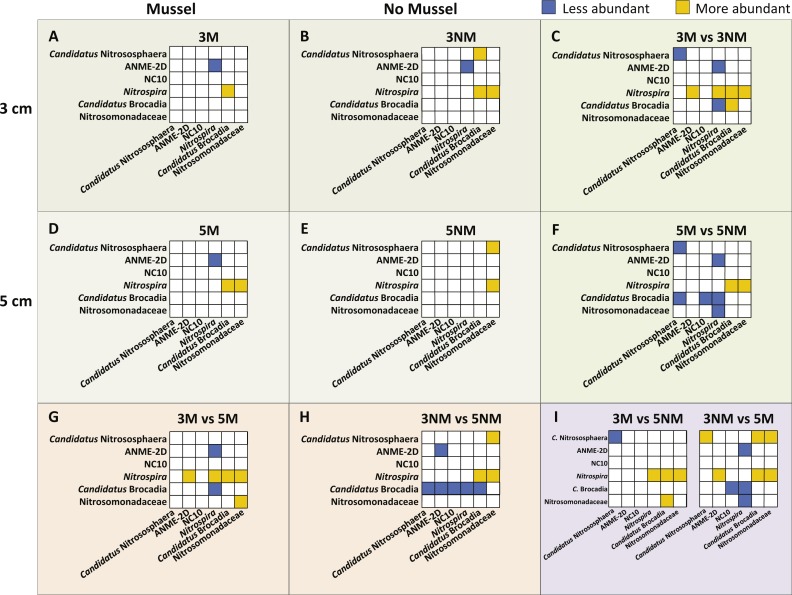
Image of N-cycle phylotype comparisons between treatments. Statistically significant differences in N-cycle organism abundances (*P* < 0.05). Statistical significance was determined by non-parametric ANOVA with Dunn’s multiple correction test. All boxes show y-axes compared to the “baseline” *x*-axes, with no boxes representing comparisons not meeting significance. (A–B), (D–E), Comparisons within treatment conditions of mussel presence and depth. (C) Differentially abundant organisms between 3 cm Mussel and 3 cm No Mussel treatments. (F) Abundance comparisons between 5 cm Mussel and 5 cm No Mussel treatments. (G) Differential N-cycle organism abundance between 3 cm Mussel and 5 cm Mussel samples. (H) Comparisons between 3 cm No Mussel and 5 cm No Mussel treatments. (I) Abundance comparisons of 3 cm Mussel versus 5 cm No Mussel, and 3 No Mussel versus 5 Mussel samples.

Relative abundances of N-cycle phylotypes were compared within each treatment (Figs. 5A, 5B, 5D, 5E) and between treatments (Figs. 5C, 5F, 5G–5I). Within 3 cm sediment samples with mussels (Fig. 5A), *Nitrospira* was statistically greater in abundance than *Candidatus* Brocadia, and ANME-2d was less abundant than *Nitrospira*. Sediment without mussels at 3 cm depth (Fig. 5B) contained statistically greater abundances of *Candidatus* Nitrososphaera than *Candidatus* Brocadia, and greater *Nitrospira* abundances compared to *Candidatus* Brocadia, Nitrosomonadaceae, and ANME-2d.

Relative abundance comparisons between mussel and no-mussel treatments at 3 cm depth (Fig. 5C) showed that *Candidatus* Nitrososphaera was reduced in the mussel treatment, while *Nitrospira* and *Candidatus* Brocadia were enhanced with mussels. Within mussel sediment samples at 5 cm depth, *Nitrospira* was more abundant than *Candidatus* Brocadia, ANME-2d, and Nitrosomonadaceae. (Fig. 5D). On the other hand, *Candidatus* Nitrososphaera and *Nitrospira* were both more abundant than Nitrosomonadaceae without mussels at 5 cm sediment depth (Fig. 5E). Comparing microbial communities at 5 cm depth between mussel and no-mussel treatments (Fig. 5F) revealed that *Candidatus* Nitrososphaera was less abundant with mussels versus the no-mussel population. *Nitrospira* and Nitrosomonadaceae phylotypes were more prominent with mussels in shallow sediment depths (Fig. 5G).

Overall, *Nitrospira* made up larger proportions of microbial communities with and without mussels compared to many N-cycle organisms, especially *Candidatus* Brocadia and Nitrosomonadaceae (Figs. 5C, 5F, and 5G–5I). Without mussels at 3 cm sediment depth, *Candidatus* Brocadia made up a smaller proportion of the N-cycling microbial community, especially when compared to *Candidatus* Nitrososphaera, ANME-2d, NC10, and *Nitrospira* in deeper sediments (Fig. 5H).

## Discussion

Numerous studies have found Proteobacteria to be the most abundant phylum in freshwater sediments (Bucci et al., 2014; Dai et al., 2016; Wakelin, Colloff & Kookana, 2008; Zeng et al., 2008; Zhang et al., 2015), sediments with mollusks (Fernandez et al., 2014; Lee et al., 2015), and also mollusk microbiomes (Frischer et al., 2000; Neta et al., 2015; Ngangbam et al., 2015; Trabal et al., 2012). Although our results showed Proteobacteria were the most abundant phylum, we observed a decrease in Proteobacteria by 6% and an increase in Nitrospirae by 10% in the presence of mussels. Families *Thermodesulfovibrionaceae* and “FW” accounted for many of the Nitrospirae OTUs that increased with mussels and helps explain decreases in species richness for mussel bed sediment.

Sediments contain the most phylogenetically diverse microbial communities (Lozupone & Knight, 2007) and structure and diversity of soil microbial communities is often determined by soil biogeochemistry (Fierer & Jackson, 2006), further supporting the impact mussels have on biogeochemical cycling. In support of our hypothesis, our data indicated that mussel presence in the UMR had a pronounced influence on the microbial community evenness, richness, and composition within the sediment. The observed changes in sediment microbial community structure and diversity showed mussels created a niche for specific microorganisms and may be attributable to the diverse chemical composition of mussel biodeposits, mixing of sediment from mussel burrowing, or the microbes living on mussels. Our findings of distinct microbial communities in mussel bed sediment are corroborated by a study of the California mussel (Pfister, Gilbert & Gibbons, 2014) where taxonomic richness increased and taxa evenness increased following the removal of mussels from a rocky shore habitat.

In contrast to our results of decreased microbial diversity with freshwater mussels, research has shown invasive zebra mussels (*Dreissena polymorpha*) increased bacterial community diversity and richness (Lee et al., 2015), and metabolic diversity and activity in freshwater sediments (Lohner et al., 2007). Increased microbial diversity and activity has been attributed to the variety of C and N components in feces and pseudofeces, and also selects for the dominant microbial species (Lohner et al., 2007; Pollet et al., 2015). An experiment combining estuarine bivalve species (*N. virens, M. arenaria, and M. balthica*) implicated mussel-induced changes in O_2_, NH_4_^+^ and NO_3_^−^ fluxes for the alteration of microbial community composition (Michaud et al., 2009). On the other hand, investigation of microbiota in Thick-shelled River Mussel (*Unio crassus*) beds did not find any difference in microorganism diversity, abundance, and composition (Richter et al., 2016). This may be explained by the drastic differences in the study site, with high mussel densities (23–433 mussels/m^2^) and control plots containing low microbial diversities with mean species richness of 48 OTUs/sample with high evenness (Richter et al., 2016). The contrasting findings of microbial community diversity and composition indicate that mussel density and/or mollusk species may produce different responses by microorganism communities.

Additionally, alterations in sediment microbial community structure may arise from exposure to the mussel shell, tissues, or fecal microbiome. Mussel tissue and fecal material has been shown to contain less diverse microbiomes than the surrounding water and sediment for the zebra mussel (Frischer et al., 2000), tropical oyster (*Crassostrea rhizophorae*) (Neta et al., 2015), and marine mussel, *Mytilus californianus* (Frischer et al., 2000; Pfister, Gilbert & Gibbons, 2014). Some studies have attributed immediate increased sediment microbial activity to the mussel intestinal microbiome (Grenz et al., 1990). Furthermore, mollusk biodeposition rates and biodeposit chemical compositions are highly dependent on mollusk species (Hegaret, Wikfors & Shumway, 2007; Tenore & Dunstan, 1973), and food availability (Bril et al., 2017; Cranford et al., 2007; Vaughn & Hakenkamp, 2001), so it makes sense that our results differ from studies with dissimilar mollusk species, densities, and study location.

Changes in mussel bed sediment microbial communities was also likely enhanced by mussel burrowing, because diffusion of substrates across the water-sediment interface is a relatively slow process (Kristensen et al., 2012) and is increased by mollusk burrowing (Vaughn & Hakenkamp, 2001), which ultimately affects microbial communities. For example, the burrow of shrimp species *Upogebia deltaura* and *Callianassa subterranean* contained distinct bacterial communities and a 3-fold increase in taxon richness (Laverock et al., 2010), and the estuarine bivalve, *C. fluminea*, stimulated microbial diversity via bioturbation (Novais et al., 2016). It is likely that UMR mussel bed sediments also experience the benefits from bioturbation, such as sediment mixing (McCall, Tevesz & Schwelgien, 1979) and aeration (Vaughn & Hakenkamp, 2001). Furthermore, bioturbation has been linked to increased NH_4_^+^ concentrations which alters the N-transforming microbial community (Chen & Gu, 2017), with greatest effects on bacteria growth found at 4–6 cm depth below the water-sediment interface (McCall, Matisoff & Tevesz, 1986).

### N-cycle microbial community

Our research revealed an increase in anammox bacteria abundance 3 cm below the water-sediment interface when mussels were present, shown for the anammox community using anammox-targeted qPCR (2.2-fold increase) and for *Candidatus* Brocadia using non-targeted 16S rRNA gene amplicon sequencing (2-fold increase). The significance of agreement between these techniques is finding that increases in the genus *Candidatus* Brocadia are representative for the anammox phylotype as a whole. *Candidatus* Brocadia may also make up a majority of the anammox community in UMR sediment, as amplicon sequencing did not detect anammox bacteria belonging to other genera. We are confident in these conclusions, as *Candidatus* Brocadia is often the dominant anammox genus in freshwater sediments (Humbert et al., 2009; Shen et al., 2016; Sonthiphand, Hall & Neufeld, 2014). One study showed that feeding of NH_4_^+^, NO_2_^−^, NO_3_^−^, and acetate led to an 80% enrichment of *Candidatus* ‘Brocadia fulgida’, signifying that B. *fulgida* could outcompete anammox species in genera *Candidatus* Anammoxoglobus and *Candidatus* Kuenenia, species *Candidatus* ‘Brocadia anammoxidans’, and even denitrifiers when acetate is present (Kartal et al., 2008). This indicates that *Candidatus* Brocadia has a distinct ecological niche and can utilize intermediates from anaerobic degradation of organic C to reduce NO_3_^−^ (Kartal et al., 2008). Therefore, it is possible that a portion of our observed increases in *Candidatus* Brocadia with mussels was attributable to C biodeposition in the UMR.

Our research also revealed a vertical distribution of anammox bacteria with higher abundances near the sediment surface, which reflects the vertical distribution found in an agricultural field (Shen et al., 2017), oxygen minimum zone (Galán et al., 2009), flooded paddy fields (Shen et al., 2017; Zhu et al., 2011), and an urban wetland (Shen et al., 2015). A vertical anammox distribution has been shown to coincide with NH_4_^+^ presence and NO_2_^−^ production (Oshiki, Satoh & Okabe, 2016; Shen, Xu & He, 2014; Shen et al., 2015; Sun et al., 2014) and anammox “hotspots” occur in zones of low, but not entirely absent, O_2_ availability (Zhu et al., 2013). Anammox abundance in freshwater sediment can range between 7 × 10^4^ and 8 × 10^6^ gene copies g^−1^ sediment (Shen et al., 2016), or between 10^6^ and 10^7^ gene copies g^−1^ sediment in peak NO_2_^−^ microniches at the oxic–anoxic interface (Nie et al., 2015; Shen et al., 2015; Zheng et al., 2016). Studies have shown anammox bacteria increase 1.5 to 2-fold within their niche (Nie et al., 2015; Zheng et al., 2016), similar to our findings of a 2.2-fold increase in anammox bacteria 3 cm below the water-sediment interfacewith mussels.

Co-occurrence of aerobic NH_4_^+^ oxidation and anammox niches are likely due to linked NO_2_^−^ oxidation and reduction, respectively (Shen, Xu & He, 2014). Interestingly, we found that mussels also enhanced taxa from the AOB family Nitrosomonadaceae and the OTUs made up a greater proportion of mussel bed sediment populations near the water-sediment interface. To this point, the pacific oyster (*C. gigas*) was found to increase porewater NH_4_^+^ and elevate the concentration of NH_4_^+^ oxidizing microorganisms (Green, Boots & Crowe, 2012). Furthermore, our previous research (Bril et al., 2014; Bril et al., 2017) showed elevated NH_4_^+^ and NO_2_^−^ in porewater of a similar depth below mussels. It makes sense that these groups of N-transforming bacteria co-occur where their substrate microniches overlap, and is likely enhanced by mussels periodically aerating the sediment (Chen & Gu, 2017). Intermittent aeration has shown to enrich microbial cultures in AOB and anammox bacteria in engineered partial nitritation-anammox processes (Shannon et al., 2015; Yang et al., 2015), and similar to our findings, enriches the anammox genus *Candidatus* Brocadia (Shannon et al., 2015).

On the other hand, we saw a decrease in *Candidatus* Nitrososphaera (AOA) with mussels at 3 cm (1.7-fold) and 5 cm (2.6-fold) sediment depths. It makes sense that mussels suppress abundance of AOA since these organisms typically dominate sediment niches with low NH_4_^+^ concentrations (Hatzenpichler, 2012; Martens-Habbena et al., 2009). Furthermore, a group of OTUs suppressed by mussels were classified at the species level as *Candidatus* ‘Nitrososphaera gargensis’, which are partially inhibited by NH_4_^+^ concentrations (3.08 mM) much lower than AOB (Hatzenpichler, 2012; Hatzenpichler et al., 2008; Nakagawa & Takahashi, 2015; Pester, Schleper & Wagner, 2011). Furthermore, nitrifier niche partitioning studies using agricultural soil showed that AOB increased in abundance and activity following the addition of urine-derived N, while AOA remained unchanged (Di et al., 2009; Hatzenpichler, 2012; Jia & Conrad, 2009). Therefore, it is possible that mussel biodeposits and an increased flux of agriculturally-fed water into sediment by mussel burrowing enhanced porewater NH_4_^+^ composition such that Nitrosomonadaceae out competed *Candidatus* Nitrososphaera. Our results agree with Chen & Gu (2017), who found bioturbated sediment corresponded with a greater diversity of AOB and lower diversity of AOA microbial communities (Chen & Gu, 2017). On the other hand, our results of decreased abundance of *Candidatus* Nitrososphaera co-occurring with an increase in *Nitrospira* is in contrast to previous findings that these organisms may exhibit similar niche partitioning (Pester, Schleper & Wagner, 2011). For example, some species in *Candidatus* Nitrososphaera can adjust their metabolism for low oxygen availability (Zhalnina et al., 2014) and *Nitrospira* species are adapted to low oxygen concentrations (Maixner et al., 2006; Nowka, Daims & Spieck, 2015; Zhalnina et al., 2014) and microoxic environments (Schramm et al., 2000). Alternatively, our detected increased abundance of *Nitrospira* may include species with a variety of environmental niches.

Some *Nitrospira* species have shown to occupy a niche at oxic–anoxic interfaces, in opposition to NOB with higher O_2_ tolerances such as those in genus *Nitrobacter* (Schramm et al., 2000). This supports our mussel-attributed increases in relative *Nitrospira* abundances (1.9-fold) at 3 cm sediment depths. Although we saw two different *Nitrospira* OTUs suppressed and enhanced by mussels, the mussel-enhanced OTUs had a larger mean abundance by about 12%. Different NOB OTUs enhanced with and without mussels further suggests that mussel bed sediments harbor specific NOB strains sensitive to microoxic niches. Despite OTU variability, we can conclude that mussels enhance the *Nitrospira* phylotype, especially near the water-sediment interface where *Nitrospira* were 1.7-times greater than deeper mussel bed depths.

On the other hand, we did not expect to see an increase in both NOB and anammox phylotypes due to competition of NO_2_^−^ as a substrate. The co-occurrence of *Nitrospira* and anammox bacteria may be explained by the metabolic versatility of *Nitrospira* species, especially if mussel-derived urea provided an additional source of NH_3_ and NO_2_^−^ via reciprocal feeding between ammonia oxidizers and *Nitrospira*. Furthermore, these phylotypes have been shown to coexist in an oxygen minimum zone, where anammox bacteria obtained a majority of NO_2_^−^ from NO_3_^−^ reducers (Lam et al., 2009). The similar effect size of mussels on *Nitrospira* (1.9-fold) and *Candidatus* Brocadia (2-fold) at 3 cm depth suggests that mussels may exert similar influences on the niches of these phylotypes. It is possible that these anammox and *Nitrospira* phylotypes were functionally linked in shallow mussel bed sediment, which has been shown for microoxic niches (Van Kessel et al., 2015). Furthermore, it is possible that the *Nitrospira* co-occurring with *Candidatus* Brocadia were *Nitrospira* species with the genetic potential for comammox, as a fluorescence in-situ hybridization study confirmed the extensive aggregation of the 2 phylotypes in hypoxic conditions (<3.1 µM O_2_) (Van Kessel et al., 2015). Despite *Nitrospira* comammox being identified in numerous aquatic environments (Chao et al., 2016; Daims et al., 2015; Pinto et al., 2016), we cannot conclusively identify comammox without sequencing the ammonia monooxygenase gene (Pinto et al., 2016; Van Kessel et al., 2015).

In contrast to studies which found significant N-reduction on both a marine mussel (*Mytilus californianus*) (Pfister, Meyer & Antonopoulos, 2010) and a freshwater mussel (*Limnoperna fortunei*) (Zhang, Cui & Huang, 2014), our results showed that mussels suppressed n-damo OTUs in phylum “NC10” (2.1-fold) and family “ANME-2d” (1.8-fold). One study determined NO_3_^−^-damo was responsible for NO_3_^−^ reduction and anammox for NO_2_^−^ reductions in a bioreactor supplied with NH_4_^+^, NO_2_^−^, NO_3_^−^, CH_4_, and anoxic conditions, thus concluding anammox outcompeted NO_2_^−^-damo (Hu et al., 2015). These findings make sense, because anammox bacteria have a higher affinity for NO_2_^−^ (Luesken et al., 2011), anammox outperform n-damo in bioturbated sediments with higher NH_4_^+^ and lower NO_2_^−^ and NO_3_^−^ (Chen & Gu, 2017), and anammox and n-damo communities have a competitive relationship in burrowed mangrove sediment (Chen & Gu, 2017). Furthermore, NC10 bacteria in a peatland were most prevalent at depths with porewater CH_4_ concentrations near 300 µM, where NO_3_^−^ consumption exceeds production, and in completely anoxic conditions (Zhu et al., 2012). According to the literature, it makes sense that we found UMR mussels enhanced *Candidatus* Brocadia and suppressed NO_2_^−^ reducing-NC10. Perhaps n-damo organisms did not have a favorable niche in mussel bed sediment because biodeposition products created an excess of NH_4_^+^ in sediment porewater (Winkler et al., 2015), or burrowing activity increased oxygen concentrations and made methane oxidation unfavorable (Van Bodegom et al., 2001). Our finding that no-mussel sediment contained three times more ANME-2d in deeper, and presumably anoxic sediment, further suggests that mussels broaden the oxic–anoxic interface niche (Chen & Gu, 2017; Luesken et al., 2011). However, we cannot extrapolate these findings to all denitrifying organisms, since denitrifying species are sporadically distributed among various taxonomic lineages, and are difficult to identify solely with16S rRNA amplicon sequencing (Ishii et al., 2011).

Although we observed greater relative abundances of *Nitrospira* than *Candidatus* Brocadia in a majority of treatments, both phylotypes increased by a factor of 2 with mussels at 3 cm depth. No-mussel samples contained a significantly smaller proportion of *Candidatus* Brocadia in shallow sediments compared to almost all N-transformers found in the deeper control sediments. Our phylotype-level analyses revealed similarities with the OTU-level differential abundance comparisons. For example, phylotype comparisons showed ANME-2d was less abundant than *Nitrospira* in 3 cm sediments with mussels, and *Candidatus* Nitrososphaera was more abundant than *Candidatus* Brocadia in 3 cm sediment samples without mussels. These results relate to DESeq2 OTU comparisons which found *Candidatus* Brocadia and *Nitrospira* enhanced with mussels while ANME-2d and *Candidatus* Nitrososphaera were suppressed with mussels.

Extending our focus beyond N-cycling organisms, we demonstrated that mussels promoted a large effect size for OTUs classified as *Thermodesulfovibrionaceae* (Nitrospirales order). In contrast to *Nitrospira*, the Nitrospirales genus *Thermodesulfovibrio* contains multiple sulfate reducing species (Kirchman, 2012; Sekiguchi et al., 2008) and can outcompete other anaerobic organisms when sulfate is present (He et al., 2015). These findings are corroborated by discoveries of significantly greater C and sulfate concentrations from mussel biodeposits and 63% greater sulfate reduction in sediments with mussels (McKindsey et al., 2011). Biodeposition products often lead to increasingly anoxic sediment and greater activity of anoxic microorganisms (Kellogg et al., 2013; McKindsey et al., 2011), presumably due to consumption of excretion products by oxygen-consuming microorganisms (Pollet et al., 2015). Interestingly, Fdz-Polanco et al. (2001) observed simultaneous N and sulfate removal in an anaerobic fluidized-bed reactor and proposed simultaneous anammox and sulfate reduction. Coupled biological sulfate reduction and anammox reactions are metabolically feasible (Schrum et al., 2009; Strous et al., 2002) and have been of interest in the recent history (Ali et al., 2013; Cai, Jiang & Zheng, 2010; Rikmann et al., 2016; Rios-Del Toro & Cervantes, 2016), therefore warranting further research. Therefore, we showed that *Thermodesulfovibrionaceae* are significantly increased in the presence of mussels which may affect sulfate reduction (Mahmoudi et al., 2015) in tandem with anammox reactions in UMR sediments.

As a whole, mussels do have an impact on microbial niches and lower the overall community diversity. Mussel-influenced changes in microbiological diversity may have larger ecosystem implications, such as macrobiota richness and diversity (Arribas et al., 2014; Borthagaray & Carranza, 2007). Native freshwater mussels are capable of increasing macrobiota diversity as a result of being keystone species (Hartmann et al., 2016) and ecosystem engineers (Chowdhury, Zieritz & Aldridge, 2016; Lopes-Lima et al., 2014). Mussel biogeochemical hotspots can lead to a bottom-up trophic cascade by enhancing N substrates normally limiting primary productivity, ultimately leading to increased richness (Atkinson et al., 2013) and biodiversity (Allen et al., 2012) of higher trophic levels.

## Conclusion

As far as we know, this is the first study to characterize freshwater mussel effects on microbial community diversity, composition, and the vertical distribution of N-cycle microorganisms in the UMR. qPCR of the anammox-specific 16S rRNA gene revealed an increase in anammox bacteria abundance 3 cm below the water-sediment interface when mussels were present, and confirmed anammox bacteria were normally distributed with depth. Non-targeted 16S rRNA gene amplicon sequencing revealed mussel presence suppressed AOA (*Candidatus* Nitrososphaera) and that the families *Thermodesulfovibrionaceae* and “FW” (Nitrospirales order) were overrepresented among the enhanced OTUs with-mussels. Mussel bed sediment contained microbial communities with 10% greater Nitrospirae and 6% fewer OTUs belonging to the phylum Proteobacteria, which ultimately had a pronounced influence on microbial community evenness, richness, and composition. This was indicated by lower observed species richness, Chao1 diversity, Shannon diversity, and clustering of mussel samples in an NMDS analysis. We have shown that native freshwater mussels affect niche differentiation of N-cycle microorganisms, as evidenced by increased abundances of AOB family Nitrosomonadaceae, anammox genus *Candidatus* Brocadia, and NOB genus *Nitrospira*, while exhibiting a decrease in AOA genus *Candidatus* Nitrososphaera, and n-damo organisms in the phylum NC10 and family ANME-2d. Co-occurring 2-fold increases in *Candidatus* Brocadia and *Nitrospira* in shallow sediment suggests that mussels may enhance microbial niches at the interface of oxic–anoxic conditions, presumably through biodeposition and burrowing. Ultimately, this study demonstrates the large impact mussels have on biogeochemical N-cycling and ecosystem services in freshwater agroecosystems.

##  Supplemental Information

10.7717/peerj.3536/supp-1 Supplemental Information 1 Supplemental information

## Additional Information and Declarations

### DNA Deposition

The following information was supplied regarding the deposition of DNA sequences: MG-RAST (ID: 4705672.3-4705709.3). Bioproject Accession: PRJNA374585.

### Data Availability

The following information was supplied regarding data availability: GitHub: https://github.com/mchimenti/black_chimenti_just_phyloseq/blob/master/phyloseq.r. Project name: Mussel1_longlistminus2. Static link: http://metagenomics.anl.gov/linkin.cgi?project=mgp18682. Metagenomes: 4705672.3 through 4705709.3. Project name: Mussel1_shortlist. Static link: http://metagenomics.anl.gov/linkin.cgi?project=mgp18674. Metagenomes: mgm4705417.3; mgm4705418.3. Individual IDs: Mussel IDs: 4705672.3, 4705673.3, 4705675.3, 4705676.3, 4705677.3, 4705680.3, 4705681.3, 4705683.3, 4705684.3, 4705690.3, 4705691.3, 4705693.3, 4705695.3, 4705696.3, 4705699.3, 4705705.3, 4705706.3, 4705708.3, 4705709.3, 4705417.3. No-mussel IDs: 4705674.3, 4705678.3, 4705679.3, 4705682.3, 4705685.3, 4705686.3, 4705687.3, 4705688.3, 4705689.3, 4705692.3, 4705694.3, 4705697.3, 4705698.3, 4705700.3, 4705701.3, 4705702.3, 4705703.3, 4705704.3, 4705707.3, 4705418.3.
