# Supplementary material for: Effect of freshwater mussels on the vertical distribution of anaerobic ammonia oxidizers and other nitrogen-transforming microorganisms in upper Mississippi river sediment
Source: PeerJ. 2017 Jul 12;5:e3536. doi: 10.7717/peerj.3536 (PMC5510576; doi:10.7717/peerj.3536)
Supplement: Supplemental Information 1 [file peerj-05-3536-s001.docx]

**Supplementary information**

**
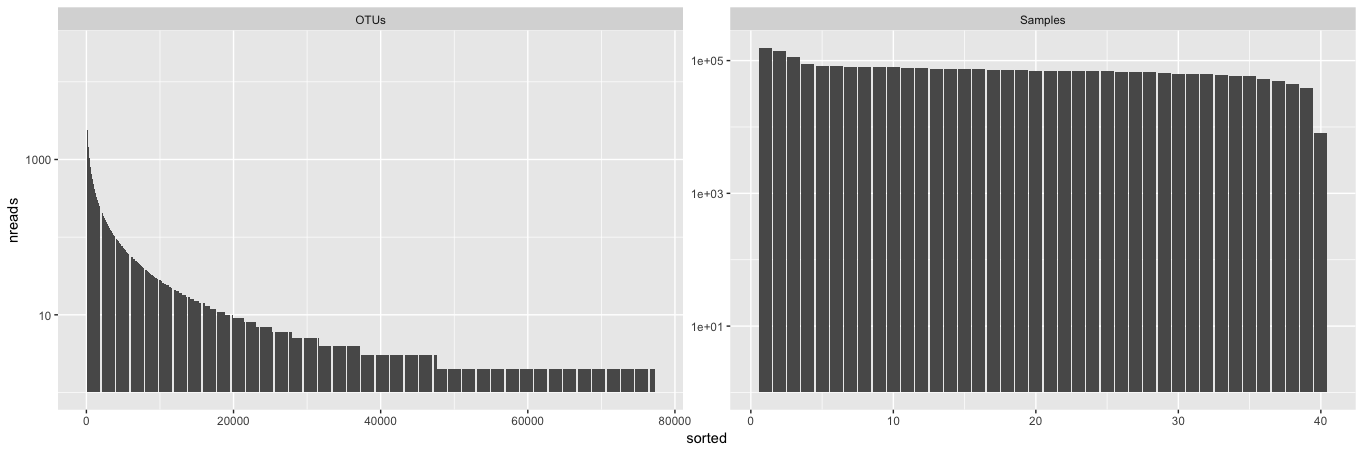
**

Figure S1: Number of reads (y-axis; log-scale) by rank-ordered OTU (left) and by sample (right) in this experiment. There were ~55,000 OTUs with fewer than 10 reads and the sample read depths were relatively consistent.


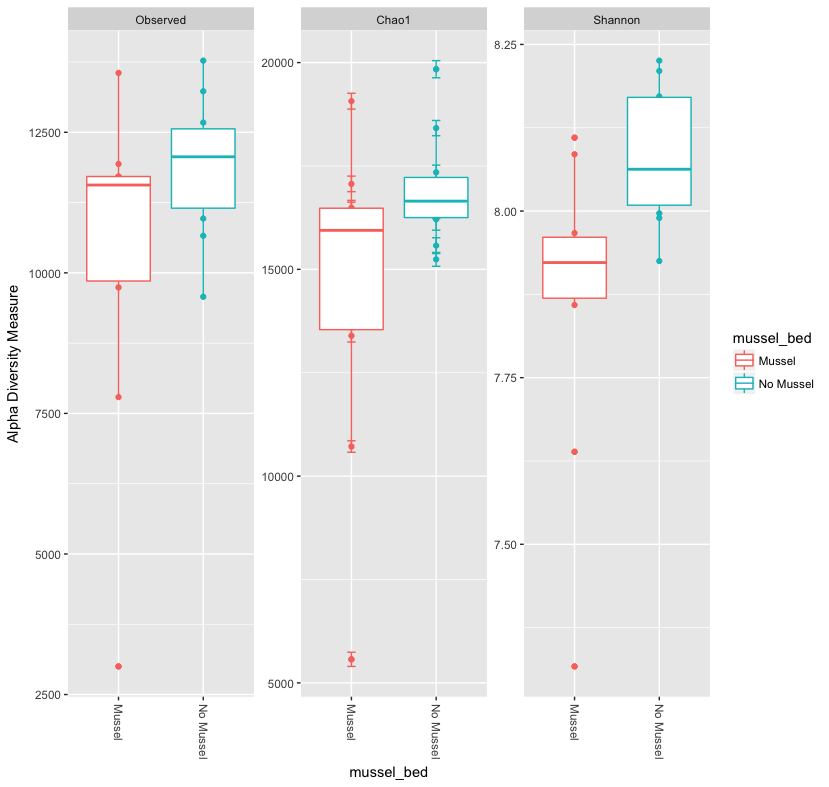


Figure S2: Alpha diversity at 3 cm depth in with-mussels and no-mussels samples.


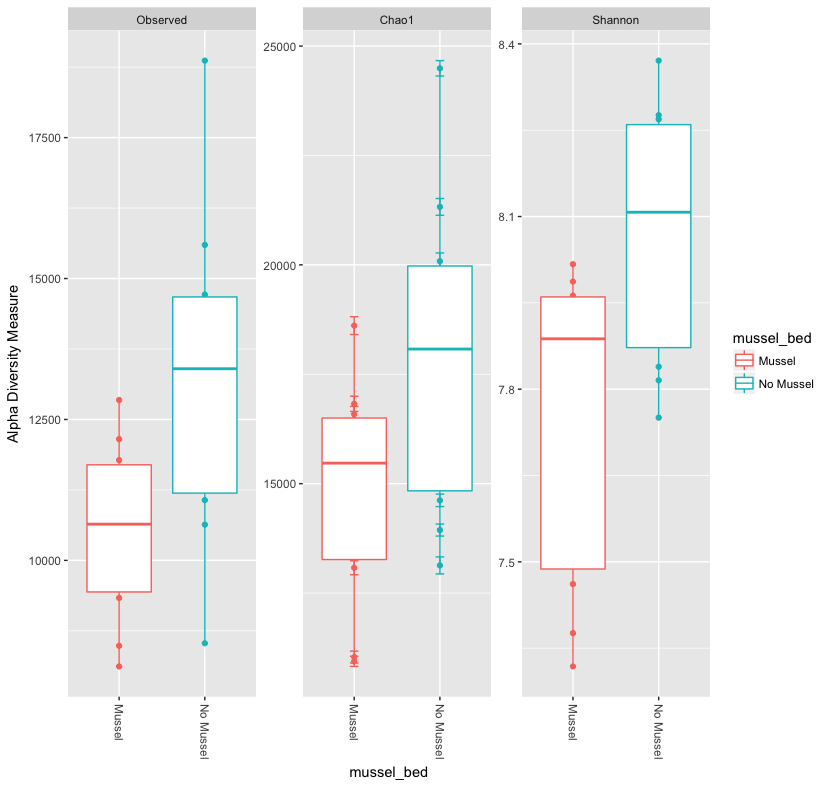


Figure S3: Alpha diversity at 5 cm depth in with-mussels and no-mussels samples.


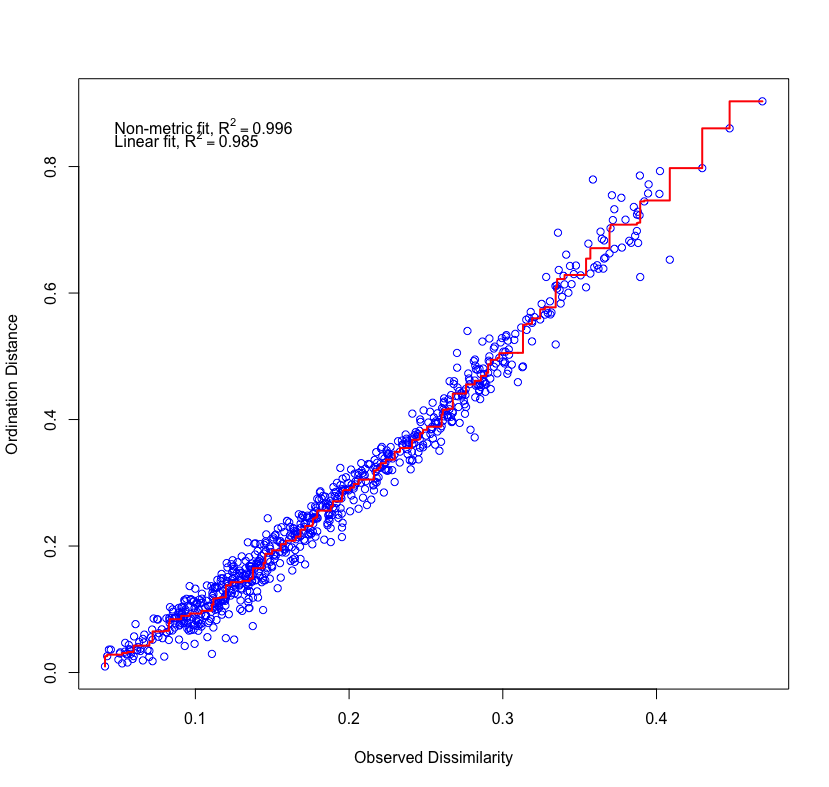


Figure S4: Shepard diagram from NMDS modeling of the sample bray-curtis distance matrix. Both the non-metric and linear fits indicate an excellent correlation to the dataset; stress converged at 0.06.

Table S1: All N-cycle OTUs with a significant Log2FC as determined by DEseq2 OTUs are listed in order of descending Log2FC.

| **Differential increases *without* mussels** | **Effect size (log2FC)** | **Average OTU count** | **Padj** | **Kingdom** | **Phylum** | **Class** | **Order** | **Family** | **Genus** | **Species** | **N-cycle classification** |
| --- | --- | --- | --- | --- | --- | --- | --- | --- | --- | --- | --- |
|  | 4.40 | 14.42 | 2.14E-08 | Bacteria | nc10 | 12-24 | Methylomirabiliales | Methylomirabiliaceae | Candidatus Methylomirabilis | unclassified | n-damo |
|  | 4.08 | 6.19 | 1.41E-04 | Bacteria | nc10 | 12-24 | JH-WHS47 | unclassified | unclassified | unclassified | n-damo |
|  | 3.88 | 2.35 | 1.88E-03 | Bacteria | nc10 | 12-24 | JH-WHS47 | unclassified | unclassified | unclassified | n-damo |
|  | 3.80 | 7.13 | 4.00E-04 | Bacteria | nc10 | 12-24 | Methylomirabiliales | Methylomirabiliaceae | Candidatus Methylomirabilis | unclassified | n-damo |
|  | 3.72 | 7.04 | 9.11E-05 | Bacteria | Planctomycetes | Planctomycetia | Candidatus Brocadiales | Candidatus Brocadiaceae | Candidatus Brocadia | unclassified | anammox |
|  | 3.59 | 21.01 | 6.80E-07 | Bacteria | nc10 | wb1-A12 | unclassified | unclassified | unclassified | unclassified | n-damo |
|  | 3.41 | 50.35 | 2.38E-06 | Archaea | Euryarchaeota | Methanomicrobia | Methanosarcinales | ANME-2D | unclassified | unclassified | n-damo |
|  | 3.32 | 3.50 | 1.49E-03 | Bacteria | nc10 | wb1-A12 | unclassified | unclassified | unclassified | unclassified | n-damo |
|  | 2.95 | 26.12 | 1.27E-04 | Bacteria | nc10 | 12-24 | JH-WHS47 | unclassified | unclassified | unclassified | n-damo |
|  | 2.93 | 33.33 | 4.92E-06 | Archaea | Euryarchaeota | Methanomicrobia | Methanosarcinales | ANME-2D | unclassified | unclassified | n-damo |
|  | 2.85 | 16.14 | 3.43E-06 | Archaea | Thaumarchaeota | Nitrososphaeria | Nitrososphaerales | Nitrososphaeraceae | Candidatus Nitrososphaera | gargensis | AOA |
|  | 2.82 | 59.13 | 1.12E-06 | Archaea | Euryarchaeota | Methanomicrobia | Methanosarcinales | ANME-2D | unclassified | unclassified | n-damo |
|  | 2.67 | 25.49 | 1.43E-05 | Bacteria | nc10 | 12-24 | JH-WHS47 | unclassified | unclassified | unclassified | n-damo |
|  | 2.08 | 129.78 | 1.98E-04 | Bacteria | nc10 | 12-24 | JH-WHS47 | unclassified | unclassified | unclassified | n-damo |
|  | 2.05 | 17.35 | 1.51E-04 | Bacteria | Nitrospirae | Nitrospira | Nitrospirales | Nitrospiraceae | Nitrospira | unclassified | NOB |
|  | 1.73 | 126.03 | 1.73E-06 | Archaea | Thaumarchaeota | Nitrososphaeria | Nitrososphaerales | Nitrososphaeraceae | Candidatus Nitrososphaera | SCA1170 | AOA |
|  | 1.66 | 21.87 | 1.39E-04 | Archaea | Thaumarchaeota | Nitrososphaeria | Nitrososphaerales | Nitrososphaeraceae | Candidatus Nitrososphaera | unclassified | AOA |
|  | 1.59 | 12.25 | 1.69E-03 | Archaea | Thaumarchaeota | Nitrososphaeria | Nitrososphaerales | Nitrososphaeraceae | Candidatus Nitrososphaera | unclassified | AOA |
|  | 1.58 | 4.50 | 6.64E-04 | Archaea | Thaumarchaeota | Nitrososphaeria | Nitrososphaerales | Nitrososphaeraceae | Candidatus Nitrososphaera | SCA1170 | AOA |

| **Differential increases *with* mussels** | **Effect size (log2FC)** | **Average OTU count** | **Padj** | **Kingdom** | **Phylum** | **Class** | **Order** | **Family** | **Genus** | **Species** | **N-cycle classification** |
| --- | --- | --- | --- | --- | --- | --- | --- | --- | --- | --- | --- |
|  | 1.88 | 5.57 | 2.26E-04 | Bacteria | Proteobacteria | Betaproteobacteria | Nitrosomonadales | Nitrosomonadaceae | unclassified | unclassified | AOB |
|  | 1.21 | 15.57 | 1.02E-03 | Bacteria | Planctomycetes | Planctomycetia | Candidatus Brocadiales | Candidatus Brocadiaceae | Candidatus Brocadia | unclassified | anammox |
|  | 0.80 | 209.27 | 1.31E-05 | Bacteria | Nitrospirae | Nitrospira | Nitrospirales | Nitrospiraceae | Nitrospira | unclassified | NOB |
